# Assessment of a non-invasive approach to pregnancy diagnosis in gray whales through drone-based photogrammetry and faecal hormone analysis

**DOI:** 10.1098/rsos.230452

**Published:** 2023-07-19

**Authors:** A. Fernandez Ajó, E. Pirotta, K. C. Bierlich, L. Hildebrand, C. N. Bird, K. E. Hunt, C. L. Buck, L. New, D. Dillon, L. G. Torres

**Affiliations:** ^1^ Geospatial Ecology of Marine Megafauna Lab, Marine Mammal Institute, Department of Fisheries, Wildlife and Conservation Sciences, Oregon State University, Newport 97365, OR, USA; ^2^ Centre for Research into Ecological and Environmental Modelling, University of St Andrews, St Andrews, UK; ^3^ Smithsonian-Mason School of Conservation, Department of Biology, George Mason University, 1500 Remount Road, Front Royal, VA 22630, USA; ^4^ Department of Biological Sciences, Northern Arizona University, 617 South Beaver Street, Flagstaff, AZ 86011, USA; ^5^ Ursinus College, 601 East Main Street, Collegeville, PA 19426, USA

**Keywords:** gray whale, progesterone, drone-based photogrammetry, enzyme immunoassay, pregnancy

## Abstract

Knowledge of baleen whales' reproductive physiology is limited and requires long-term individual-based studies and innovative tools. We used 6 years of individual-level data on the Pacific Coast Feeding Group gray whales to evaluate the utility of faecal progesterone immunoassays and drone-based photogrammetry for pregnancy diagnosis. We explored the variability in faecal progesterone metabolites and body morphology relative to observed reproductive status and estimated the pregnancy probability for mature females of unknown reproductive status using normal mixture models. Individual females had higher faecal progesterone concentrations when pregnant than when presumed non-pregnant. Yet, at the population level, high overlap and variability in progesterone metabolite concentrations occurred between pregnant and non-pregnant groups, limiting this metric for accurate pregnancy diagnosis in gray whales. Alternatively, body width at 50% of the total body length (W50) correctly discriminated pregnant from non-pregnant females at individual and population levels, with high accuracy. Application of the model using W50 metric to mature females of unknown pregnancy status identified eight additional pregnancies with high confidence. Our findings highlight the utility of drone-based photogrammetry to non-invasively diagnose pregnancy in this group of gray whales, and the potential for improved data on reproductive rates for population management of baleen whales generally.

## 1. Introduction

The ability to quantify the number of pregnancies within a population provides valuable knowledge about the health of individual females and the population as a whole [1,2]. Additionally, reproductive information facilitates evaluation of other critical life-history parameters, including the age of sexual maturity, inter-calving interval, frequency of pregnancy, duration of gestation, phenology of reproduction and population fecundity, all of which are essential components for monitoring trends in reproduction and the overall health of a species [3].

Unfortunately, the reproductive physiology of baleen whales is among the least understood of all vertebrates [4,5]. The large size, cryptic behaviour and relatively inaccessible habitat of large cetaceans pose limitations to the application of physiological methods traditionally used to identify pregnancy in terrestrial or smaller aquatic vertebrates. Moreover, large whales have few definitive external indicators of the reproductive state beyond the presence of a dependent calf [6]. Owing to the inability to safely capture and handle these large animals [3,5], observational data are difficult to pair with physical examinations or circulating hormone concentrations. Hence, detailed knowledge of the life-history and general reproductive biology of whales is sparse for most species and populations. In fact, much of the available information is derived from whaling records [7], which may be outdated for application in population models [8]. Fortunately, in recent years, researchers have validated using innovative sample types (i.e. alternatives to blood) to inform estimates of physiological status in live whales. For example, steroid hormone data derived from blubber biopsy specimens [8–11], respiratory vapour [12,13] and faeces [4,13–18] have proven relevant for addressing questions of stress and reproductive physiology in baleen whales. However, in most cases, these samples can only measure a single point in time in the whale's life, and repeated sampling of the same individual is often not possible or, in the case of certain sample types, may cause undesirable disturbance or even small wounds (e.g. biopsies).

Steroid hormones (i.e. progestins, oestrogens, androgens, glucocorticoids and mineralocorticoids) are the principal mediators of reproduction and the stress response in mammals. For this reason, they are widely employed as biomarkers of stress and reproduction in terrestrial and aquatic wildlife, including cetaceans [3,14]. Steroids are cleared from the blood primarily by the liver, evacuated into the gut via the bile ducts, modified by gut microbiota to produce a family of related ‘faecal hormone metabolites’, and excreted in faeces, with another portion excreted via the urine [15–17]. Faecal hormone metabolites can be quantified in faeces using antibodies that bind to the parent hormone and that, ideally, also have a high cross-reactivity with some, or all, of the common mammalian faecal metabolites of that hormone [18,19]. The metabolic clearance rates and intestinal transit time for a species determines the latency between the hormone being secreted into the blood and peak hormone excretion in faeces [16,20]. In large mammals, this lag-time between secretion and excretion is typically between 1 and 2 days [16,17,21,22]. Several decades of validation studies for both terrestrial and marine vertebrates have verified that, in general, quantification and analysis of faecal hormone metabolites provides a non-invasive technique which allows researchers to repeatedly sample individuals for several steroid hormones. The quantification of these steroid hormones is known to be highly relevant for assessing reproduction and stress [5,21,23].

Progesterone, colloquially known as the ‘pregnancy hormone’ due to its fundamental role in establishing and sustaining pregnancy in most mammals, is of particular interest to understanding whale reproduction [24,25]. In most mammals, circulating progesterone in pregnant females is higher, often by orders of magnitude, than in non-pregnant females [26]. Such pronounced differences in concentrations have enabled validation of faecal progesterone metabolites (fP4m) as a biomarker of pregnancy in various cetacean species including the North Atlantic right whale (*Eubalaena glacialis*; [27,28]), humpback whale (*Megaptera novaeangliae*; [29]), blue whale (*Balaenoptera musculus;* [30]) and gray whale (*Eschrichtius robustus*; [22]). However, analysis of progesterone concentrations for pregnancy diagnosis presents challenges. For example, if a pregnant individual is sampled at the beginning of gestation, a spurious low result may arise if progesterone progressively increases throughout gestation in cetaceans, as occurs in many other mammals [31]. By contrast, if the sampling effort is concentrated towards the end of the gestation period, pregnancies that were terminated early may be undetected. Consequently, pregnancy diagnosis based on fP4m alone might not be sufficient to provide an accurate assessment of an individual's reproductive status.

Unoccupied aerial systems (UAS, a.k.a. drones) offer an alternative and complementary source of data relevant to pregnancy diagnosis in large whales. Drones have been effectively employed to assess whale body morphology and infer aspects of physiological state [32–38]. Specifically, photogrammetry of images collected with drones that are calibrated with altitude data [33,36,39] can be used to accurately measure body length and widths, which can help assess individual and population health [18,37,39], and can estimate maternal energetic investment in offspring [32,40]. Even prior to the application of drones to study whale morphology, aerial images collected from manned aircraft have been used to estimate reproductive status in adult female right whale (*Eubalaena* sp.; [41]), and images from gray whales during their southbound migration revealed suspected near-term pregnancies in whales that were wider, relative to length, than others [42]. Drone-based photogrammetry has helped identify reproductive status and pregnancy in bottlenose dolphin (*Tursiops truncatus*; [43]). In baleen whales, pregnant females are comparatively larger than individuals in other demographic units (e.g. humpback whale [44] and gray whale [35]). The use of drones in large whale field programmes has proliferated over the past decade [45]. As a result, aerial images that could be used to identify pregnancy (e.g. from abdominal contour) are routinely being collected, yet the analytical methods needed to diagnosis pregnancy need further development.

Improved methodology for pregnancy diagnosis would be particularly useful for gray whales, which have suffered several recent unusual mortality events (UMEs). Gray whales in the Eastern North Pacific (ENP) undergo annual migrations between their southern wintering grounds in Baja California, Mexico, and their northern feeding grounds in the Bering and Chukchi seas in the Arctic. The annual calf production for this population has been consistently monitored through shore-based counts of female gray whales accompanied by calves (i.e. mother–calf pairs) since 1994 [46,47]. Records indicate periods of low calf production that align with periods of UMEs (1999–2000 and 2019–2022) and declines in abundance [47,48]. Although these calf count methods provide estimates of calf production, important information on pregnancy rates and loss is unmonitored, which is essential data for studying mammalian population dynamics [43]. Hence, the combination of fP4m and drone-based photogrammetry for pregnancy diagnosis in gray whales could improve assessments of populational reproductive biology. Yet, to our knowledge, the combination of such non-invasive approaches for pregnancy diagnosis has not been applied in large whales.

Validation of faecal hormones and drone-based photogrammetry to accurately diagnose pregnancy and monitor reproductive dynamics requires a long-term individual-based study that provides the required life-history information, i.e. age, sex and confirmed reproductive status. Among ENP gray whales, a distinct group of approximately 200–250 individuals [49] recognized as the Pacific Coast Feeding Group (PCFG; [7,50,51]), provides a unique opportunity to test, validate and compare both of these potential pregnancy-diagnosis methods. These whales forage along the Pacific coast from northern California, USA to British Columbia, Canada during the summer, usually remaining within 10 km of shore [52]. The female gray whale reproductive cycle is typically 2 years [53]. Conception occurs during the southbound migration (i.e. December–January) and gestation lasts approximately 13 months, with most births occurring during the month of January. The lactation period lasts around seven months, usually ending in August [53]. PCFG gray whales display strong site fidelity, which allows for high individual resighting rates [54]. Photo-identification of the PCFG has been conducted for over 30 years by multiple research groups that contribute data to a photo-ID catalogue curated by the Cascadia Research Collective (Olympia, WA, USA), enabling age estimation of individuals. Further, sex identification of PCFG whales is often available through genetic sampling [22,55] and observation records of females with calves.

Therefore, the PCFG is an excellent study group to develop and advance non-invasive methods of pregnancy diagnosis for baleen whales. We conducted six consecutive years of field effort in nearshore waters of Oregon, USA collecting data on PCFG gray whales to evaluate the utility of combining faecal hormone analyses with drone-based photogrammetry to identify pregnancy. Specifically, we set out to (i) explore the variation in fP4m and body morphometrics in relation to the observed reproductive status in gray whales, and (ii) develop and compare models to classify females of unknown reproductive status using an expectation–maximization (EM) algorithm to fit normal mixture models to the data derived from these non-invasive techniques. Given the inherent challenges of physiological studies of baleen whales, non-invasive diagnosis of pregnancy could significantly advance conservation management efforts of these threatened and protected animals through assessment of reproductive effort, success and loss.

## 2. Material and methods

### 2.1. Sampling location and field methods

We conducted sampling efforts from a small rigid-hulled inflatable boat (5.4 m) during the PCFG foraging seasons (late May to mid-October) along the central Oregon coast, USA (off Newport, 44°38′13″ N, 124°03′08″ W) annually from 2016 to 2021. Once a gray whale or whale group was located, we photographed individuals for identification purposes and conducted drone flights for photogrammetry analysis (details below) as weather conditions allowed. We opportunistically collected faecal samples at whale sightings using two dipnets outfitted with 300 µm nylon mesh; samples were immediately transferred to sterile plastic jars, placed on ice, and then stored in a freezer (−20°C) upon returning from the field within 2–6 h after collection. By contrast to faecal samples from some other species (e.g. North Atlantic right whale), faeces of gray whales in this study area consist of small particles that diffuse and sink quickly in the water column; thus, the amount of faecal material collected varied among samples depending on not just defecation mass, but also on environmental conditions and speed of sampling (e.g. how quickly dipnet collection began after defecation), which itself was contingent upon avoiding the whale's path. We performed hormone analysis within 11 months of collection (details below). We documented date, time and location for each faecal sample and linked these data to specific individuals via photo-identification matching [56,57].

### 2.2. Drone-based photogrammetry

We collected aerial videos of PCFG gray whales using drones (electronic supplementary material, table S1). We recorded videos at a minimum altitude of 25 m. We did not observe any behavioural responses of whales to the UAS (i.e. no change of direction, sudden dive, increased swimming speed, etc.). We extracted snapshots of individual whales from the aerial videos using VLC Media Player (v. 3.0.16; VideoLAN, Paris, France) for photogrammetry analysis. We measured the total body length (TL, measured as snout to fluke notch) and body width, in 5% increments between 20% and 70% of the whale's TL, using MorphoMetriX [58] and then processed using CollatriX [59]. Following our published methodology for this species, we then standardized the body widths by TL, which produces a scale-invariant and unitless metric that allows comparison across individuals with high precision [39]. All UAS are susceptible to photogrammetric uncertainty associated with the altimeter, camera, focal length and pixel measurement [36]. To incorporate this uncertainty associated with each UAS, we applied Bayesian methods to generate a posterior predictive distribution for each morphological measurement [36,39]. We used measurements of a 1.0 m wooden board floating at the surface in images collected between 20 and 70 m altitude as our calibration object and training data for the Bayesian statistical model [36].

### 2.3. Individual photo-identification: age, sex, and reproductive state

We used Adobe Bridge (v. 8.0.1.282) to assess whale identification photographs, using only high-quality images that were in focus and not affected by glare, angle or distance [56,57]. Sex was determined based on (i) observation (i.e. mother with a calf), (ii) previous genetic analysis of tissue samples for individuals identified from the photo-ID catalogue [60], or (iii) from faecal sample genetics analysis [35]. We estimated the ages of individuals based on the length of their sighting history (LSH) from the photo-identification catalogue. Individuals first observed as calf were considered to have a ‘known age’ equal to the LSH, whereas individuals not first observed as a calf were considered to have a ‘minimum age’ equal to the LSH.

For this study, we investigated known female whales (*n* = 51 individuals) assigned into one of four reproductive classes: juvenile female (JF), mature female (MF), pregnant female (PF) and lactating female (LF). Individuals were classified as ‘mature’ if their known age or minimum age was more than or equal to 8 years (i.e. MF), which is the mean age of sexual maturity for gray whales based on histological examinations of gonads and lamina of earplugs [7,61], individuals with a known age less than 8 years were classified as ‘juveniles’ (i.e. JF). Individuals with an unknown age (no sightings history) or a minimum age less than 8 years, were classified as mature if greater than 50% of their the estimated TL from posterior predicted distribution (see above), was greater than 11.7 m, which is the average length at maturity for female gray whales [7,36], and as juveniles if less then or equal to 50% of their estimated TL was less than 11.7 m. Females sighted with a calf at the time of sample collection were classified as LF, and whales observed with a calf the year after sample collection were presumed to be pregnant at time of faecal collection and classified as PF. We considered all immature and lactating females as presumed non-pregnant (lactation period lasts approx. seven months, and there are no known cases of lactating gray whales producing a new calf the next year). We classified as MF any mature females of unknown pregnancy status; this group is assumed to include a mixture of pregnant and non-pregnant whales. Therefore, we did not include the MF group in the initial analyses to develop pregnancy diagnostic statistical methods.

### 2.4. Faecal hormone metabolites

Faecal samples from 2016 to 2018 were previously analysed by Lemos *et al*. [22]. In subsequent years (2019–2021), we followed the same protocols for hormone extraction and quantification. Briefly, we filtered, desalted (to remove spurious inflation of dried faecal mass by salt crystals) and freeze-dried the faecal samples. We weighed the dried and homogenized samples to the nearest 0.1 mg, and excluded samples below 0.02 g from the analysis to avoid inflated values (‘small sample effect’; see [4,62]). Faecal samples contain the metabolized breakdown products of progesterone; we used a progesterone assay kit whose antibody was originally raised against progesterone, but that also cross-reacts to 5α-reduced progestin metabolites (#ADI-900-011), and thus our metric quantifies a subset of faecal progestin metabolites (fP4m). Specifically, the manufacturer reports cross-reactivities of 100% to 5a-pregnane-3,20-dione, 3.46% to 17-OH-progesterone, 1.43% to 5-pregnen-3b-o1-20-one, and less than 1% for all other tested steroids (https://www.enzolifesciences.com). We extracted the fP4m from the aliquoted faecal sample with 90% methanol (Methanol HPLC grade, Fisher Chemical), maintaining the sample mass to solvent volume ratio within a range of 1 : 10 to 1 : 25 and vortexing at room temperature for 30 min at 500 r.p.m. Then we centrifuged the mixture (sample and methanol) at 2200 r.p.m. for 20 min to separate the pellet from the supernatant with extracted hormones. We dried down the supernatant under vacuum and then we reconstituted the extracted hormones in deionized water with sonication and vortexing (1 : 1 dilution). We quantified the fP4m using a commercial Enzyme-linked Immunosorbent Assay kit for progesterone (#ADI-900-011) from Enzo Life Sciences, following the manufacturer's protocols (https://www.enzolifesciences.com). Finally, we converted the raw data (pg ml^−1^) to ng of hormone per g of dried faeces correcting by the volume of extraction and the dilution factor used when applicable. This kit has been successfully used for pregnancy diagnosis using blubber samples of odontocetes [63]; blubber, serum and urine of bowhead whale (*Balaena mysticetus*) [64]; blubber of blue whale [65] and faeces of some terrestrial artiodactyls [66]. For quality assurance and quality control, we run all samples in duplicate, including a full standard curve (i.e. six standards with a concentration range from 500 to 15.62 pg ml^−1^), and an internal control (i.e. a progesterone standard of known concentration) in each assay. We reran any samples with greater than 15% coefficient of variation (CV) between replicates, and, if the sample fell outside of the per cent-bound range of 15–98%, we adjusted the dilution accordingly and reanalysed the sample. For the values below the limit of detection (less than LOD), we assigned a concentration of half the LOD reported by the manufacturer (i.e. LOD = 8.57 pg ml^−1^ according to the information reported by the manufacturer for the Progesterone EIA kit #ADI-900-011, https://www.enzolifesciences.com). When we collected more than one sample from one individual on the same day, we combined the samples into a single jar prior to analysis, except for a few cases (*n* = 6), where the whale ID was not confirmed for the faecal sample while in the field. We analysed these six samples separately; once it was determined (from photo-identification) that they were duplicate samples (i.e. another sample had been collected from that whale on the same day), in the analyses we included only the sample with higher faecal mass. The progesterone assay kit used in this study has previously been validated for gray whale faecal samples with satisfactory parallelism and accuracy [22].

### 2.5. Data analysis

We sought to determine whether the faecal progesterone and photogrammetric techniques were viable tools for non-invasive diagnosis of pregnancy in gray whales. To this end, we explored the data with univariate and multivariate normal mixture models to assign probabilities of pregnancy for females with unknown reproductive status. Based on the evidence that gray whales conceive during the southbound migration [7,53], we assume that all faecal samples and morphometric measurements from pregnant females were collected at around six to nine months of gestation (gestation length approx. 13 months [7,53]). When multiple faecal samples were obtained from an individual in a given year, we reported and analysed the median apparent fP4m concentration for that year under the assumption that the median fP4m values would more accurately reflect the reproductive condition of each individual in a given season [31]. Similarly, when multiple photogrammetric measurements were collected from an individual in a given year, we analysed the maximum widths that an individual reached each year under the assumption that pregnant females will become wider over time. Furthermore, we only used photogrammetry measurements taken in the late season (after 26th August) each year. This late season cut-off was chosen to avoid including individuals in the early season that have recently arrived from their wintering lagoons after fasting, while still capturing most of the measurements available from the known pregnant females in our dataset. Only one PF (Er-0333, observed on 7 July 2021) was excluded using this cut-off. Nine other faecal samples were excluded from the analyses because either we could not reliably match the sample with an individual whale (*n* = 3), or because the sample came from an unknown individual with unknown sex or unknown maturity status (*n* = 6). Our final dataset consisted of 76 fP4m observations (including PF = 5, LF = 4, MF = 48 and JF = 19), and 77 morphometric measurements (including PF = 5, LF = 3, MF = 52 and JF = 17). In 42 cases, we obtained both fP4m and morphometric measurements from the same individual in the same year (including PF = 5, LF = 2, MF = 25 and JF = 10; electronic supplementary material, table S2). fP4m values were log-transformed to ensure that they were normally distributed, and normality was assessed visually with a normal Q-Q plot of the residuals (electronic supplementary material, figure S2).

As part of exploratory data analysis, we investigated how fP4m and the morphometric variables were distributed according to presumed pregnancy status (i.e. pregnant females (PF) versus presumed non-pregnant females (JF and LF)) and performed a one-way ANOVA, with a *post hoc* Tukey honestly significant difference (HSD) test for multiple comparisons (see electronic supplementary material, figures S1 and S2 for model's assumptions tests). We visually determined that the width at 50% of the total length (W50) was the morphometric variable that provided the best separation between the two groups, with the least overlap and dispersion (see Results, figure 3). The W50 also falls around the maximum body width of a gray whale's profile, which has been proposed as a good variable for recognizing near-term pregnant gray whale females along their southbound migration [42]; therefore, only this morphometric variable was included in subsequent analyses.

We used Monte Carlo methods to propagate photogrammetric uncertainty by averaging the results of 80 000 replications of an ANOVA comparing W50 by female demographic unit (JF, LF, PF) [37]. For each replicate, we sampled each whale's W50 from a normal distribution parametrized with the posterior mean and variance from that whale's posterior distribution [44] and calculated the difference between the coefficients for W50 for each demographic unit. We then calculated the mean and highest posterior density intervals (HPDI) for the difference between each demographic unit.

We used the EM algorithm to fit a multivariate normal mixture model (R package: mixtools) to the log-transformed fP4m and W50 data from all females (Model 1). We assumed that the mixture had two components: one component characterized by low fP4m and low W50 (presumed non-pregnant females) and one by high fP4m and high W50 (presumed pregnant females). Therefore, the posterior probability of pregnancy for each combination of fP4m and W50 values in the data was then calculated as the ratio of the probability density for the component with higher means for the two variables to the sum of the two probability densities. We use a non-parametric bootstrap approach, similar to the one applied in Melica *et al*. [8], to quantify the uncertainty around each probability estimate. Specifically, we resampled the variables with replacement 10 000 times, fitted the mixture model to the bootstrapped dataset, and estimated the probabilities of pregnancy for all combinations of values in the data. Owing to the small number of data points from pregnant females, those records were included in all bootstrap samples [8]. We calculate the 95% confidence intervals using the 2.5th and 97.5th percentiles of the estimated probabilities of pregnancy. We also used the EM algorithm to fit univariate normal mixture models to each of the two variables separately. For fP4m (Model 2), we fitted a normal mixture model with two components, with the assumption that these would capture two groups of individuals characterized by either low (presumed non-pregnant) or high (presumed pregnant) fP4m, and similarly, for W50 we first fitted a model with two components (Model 3) assuming that the model would capture two groups of individuals characterized by either low (presumed non-pregnant) or high (presumed pregnant) W50.

For each model, we only retained bootstrap samples if the mixture model identified both variables as having a greater mean for the component assumed to correspond to pregnant females (e.g. high fP4m or high W50); and we discarded non-convergent models [8]. Under these conditions, only 65% of the bootstrap samples were retained for the Model 1 and greater than 95% of the bootstrap samples were retained for the two univariate models (Models 2 and 3). We used the bootstrap procedure described above to calculate the probability of a female whale belonging to the component with high fP4m or high W50 (median and 95% confidence intervals). We compared output of the three models in terms of their ability to classify whales of known reproductive status, considering that the model classified an individual as pregnant when the assigned probability was greater than 75% (model performance, table 3). Further, we applied the three models to assign a pregnancy probability to all MF and evaluated the agreement among these classifications. We conducted all statistical analyses in R, and tested mean comparisons between the PF and non-pregnant (LF and JF) groups with a significance level of 0.05.

## 3. Results

### 3.1. Exploratory analyses

#### 3.1.1. Field-observed reproductive status relative to faecal progesterone metabolites fP4m

**Figure 1.**
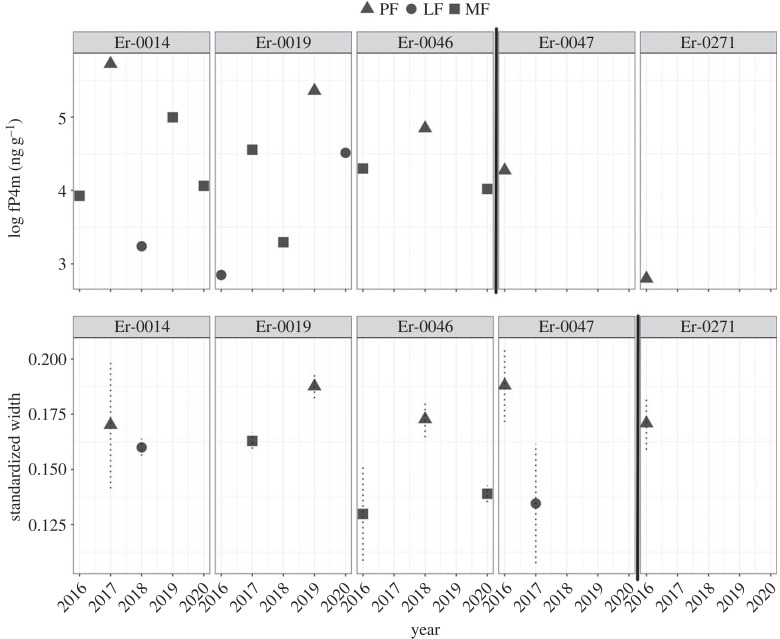
Individual variation in log-transformed faecal progesterone metabolite concentrations (in ng of immunoreactive hormone per g dried faeces; top) and standardized width at 50% of total body length, with uncertainty represented as dashed lines, as 95% highest posterior density intervals (bottom), for females that had at least one confirmed pregnancy. Note that whales Er-0047 and Er-0271 only have fP4m data during pregnancy; these individuals are included to highlight the observed ranges of fP4m and W50 for the pregnant group. The black solid vertical line separates the individuals with observations in multiple reproductive states (on the left) and the ones with observations only when pregnant (on the right).

**Figure 2.**
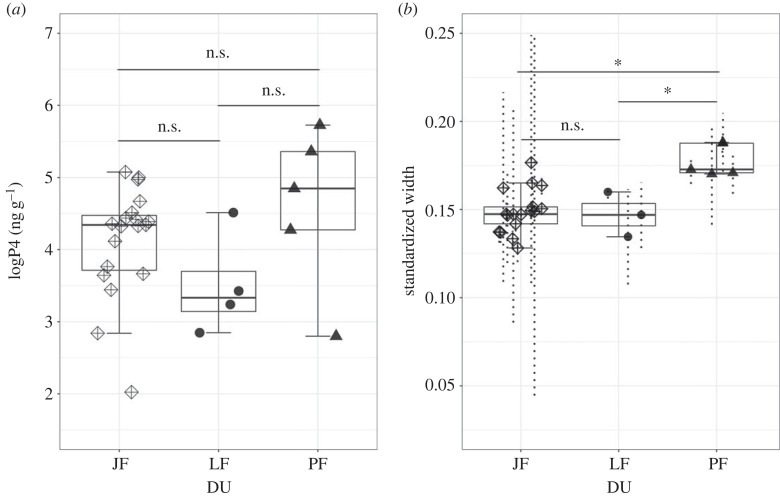
Group mean comparisons for log-transformed faecal progesterone metabolite concentrations (in ng of immunoreactive hormone per g dried faeces (*a*), and standardized width at 50% of total body length (*b*) between presumed pregnant (PF = females seen with a calf the year after sampling) and presumed non-pregnant females (LF = lactating females observed with a calf the year of sampling and JF = sexually immature females). The black horizontal lines represent the group mean; box encloses 50% of the data; whiskers enclose the smallest and largest values within 1.5 times interquartile range below and above the 25th and 75th percentile, respectively; individual values shown as circles, with uncertainty in morphometric measurements represented as dashed lines, as 95% highest posterior density intervals); n.s. denotes no statistical difference between the group means, while asterisks denote a significant difference based on the ANOVA test.

At the individual level, we observed that females resighted in multiple years with different presumed reproductive statuses (*n* = 3) exhibited elevated fP4m values when presumed pregnant (PF) as compared with when presumed not pregnant (LF and MF; figure 1). However, the observed fP4m range for the PF group (mean = 4.60, s.d. = 1.15 ng g^−1^) overlapped with the observed range for the presumed non-pregnant groups (i.e. JF mean = 4.10, s.d. = 0.77 ng g^−1^; LF mean = 3.49, s.d. = 0.62 ng g^−1^; table 1 and figure 2*a*), and at a population level, we did not detect statistical differences between the group means for presumed pregnant and presumed non-pregnant females (ANOVA test: *p*-values >0.05; figure 2*a*). The observed overlap in fP4m range is largely driven by two individuals (Er-0047 and Er-0271; figure 1 and table 2) sampled in 2016 and observed in 2017 accompanied by a calf. These two individuals were not resampled in any other year (figure 1). In six instances, two samples were obtained from the same individual on the same day (three MF and three JF) and analysed separately. We report a coefficient of variation (CV) between these repeat samples as ranging from 12 to 35 for the MF groups and 0.36 to 65 for the JF group.

**Table 1.** Descriptive statistics of potential pregnancy indicators: mean (range) of the log of faecal metabolites of progestins (fP4m, in ng of immunoreactive hormone per g dried faeces) and standardized width at 50% of total body length (W50) by presumed reproductive status; PF: observed with a calf after the year of sampling, LF: observed with a calf the year of sampling, JF: immature female, MF: adult female not seen or not seen accompanied by a calf in the year after sample collection.

reproductive status	mean fP4m (range)	mean W50 (range)
pregnant female (PF)	*n* = 5; 4.60 (2.80–5.73)	*n* = 5; 0.18 (0.17–0.19)
lactating female (LF)	*n* = 4; 3.49 (2.85–4.51)	*n* = 3; 0.15 (0.15–0.16)
juvenile female (JF)	*n* = 19; 4.22 (2.84–5.08)	*n* = 17; 0.15 (0.13–0.16)
mature female (MF)	*n* = 48; 4.05 (2.30–6.11)	*n* = 52; 0.15 (0.12–0.18)

**Table 2.** Posterior probability of pregnancy **P(p)** for each combination of fP4m and W50 assigned to each female gray whale of known and unknown reproductive status via bootstrapping (*N* = 10 000). For each whale, **P(p)** was calculated based as the ratio of the probability density for the component with higher means for the two variables to the sum of the two probability densities for each model, and the lower (LQ), and upper (UQ) quartiles are reported. Additional information included year, age, age type, log-transformed faecal progesterone metabolites (fP4m), standardized maximum width at 50% of total body length in the late season (W50) and sighting history (RS: resighted in the following year, calf: resighted with a calf, and date: earliest date seen in the following year). Individuals with a **P(p)** > = 70% are bolded. Note: LSH, length of sighting history in years; n.a., not available, i.e. data were not available to estimate a probability, or no sighting information was available; unk*, whale Er-0019 was reported in the area in 2021, although not during our field efforts and the exact date of the sighting is unknown; RS, resighted the following year; calf, ‘yes’ if resighted with a calf / ‘no’ if no calf was seen with the female; LQ, lower quartiles 25th; UQ, upper quartiles 75th. TL, total body length from snout to fluke notch expressed in metres, in parenthesis (s.d.) is the standard deviation of the posterior distribution for TL [36].

whale ID	year	LSH	age type	TL (s.d.)	fP4m	W50	Model 1			Model 2			Model 3			sighting history		
							**P(p)**	LQ	UQ	**P(p)**	LQ	UQ	**P(p)**	LQ	UQ	RS	calf	date
presumed pregnant females																		
Er-0019	2019	23	min age	12.36 (0.17)	5.36	0.19	**1.00**	1.00	1.00	**1.00**	0.40	1.00	**1.00**	0.98	1.00	yes	yes	29 June 2020
Er-0014	2017	22	known age	11.97 (0.10)	5.73	0.17	**1.00**	1.00	1.00	**1.00**	0.09	1.00	**0.92**	0.73	1.00	yes	yes	20 June 2018
Er-0046	2018	32	min age	12.83 (0.10)	4.85	0.17	**1.00**	0.99	1.00	**1.00**	0.47	1.00	**0.96**	0.80	1.00	yes	yes	9 June 2021
Er-0047	2016	23	min age	12.81 (0.37)	4.27	0.19	**1.00**	0.98	1.00	**1.00**	0.55	1.00	**1.00**	0.98	1.00	yes	yes	19 July 2017
Er-0271	2016	18	min age	11.76 (0.24)	2.80	0.17	0.00	0.00	0.00	0.05	0.00	0.22	**0.93**	0.77	1.00	yes	yes	5 June 2017
lactating females																		
Er-0211	2016	21	min age	12.32 (0.32)	3.43	0.15	0.04	0.00	0.30	0.54	0.01	0.97	0.01	0.00	0.36	yes	no	30 June 2017
Er-0014	2018	23	known age	11.97 (0.10)	3.24	0.16	0.01	0.00	0.05	0.32	0.00	0.90	**0.87**	0.26	0.99	yes	no	16 July 2019
Er-0019	2016	20	min age	12.36 (0.17)	2.85	n.a.	n.a.	n.a.	n.a.	0.05	0.00	0.21	n.a.	n.a.	n.a.	yes	no	6 June 2017
Er-0019	2020	24	min age	12.36 (0.17)	4.51	n.a.	n.a.	n.a.	n.a.	**1.00**	0.57	1.00	n.a.	n.a.	n.a.	yes	no	unk*
Er-0047	2017	24	min age	14.69 (0.59)	n.a.	0.13	n.a.	n.a.	n.a.	n.a.	n.a.	n.a.	0.00	0.00	0.50	yes	no	22 June 2018
juvenile females																		
Er-0358	2019	3	min age	9.18 (0.32)	4.51	0.18	**1.00**	0.98	1.00	**1.00**	0.56	1.00	**0.98**	0.90	1.00	yes	no	28 Aug 2020
Er-0252	2019	5	known age	10.83 (0.10)	5.00	0.16	**1.00**	0.96	1.00	**1.00**	0.63	1.00	0.62	0.30	0.99	yes	no	8 Mar 2020
Er-0318	2019	5	min age	9.99 (0.10)	4.44	0.16	**0.93**	0.79	1.00	**1.00**	0.57	1.00	0.53	0.23	0.99	yes	no	21 June 2020
Er-0377	2019	5	known age	11.05 (0.24)	4.36	0.17	**0.93**	0.78	1.00	**1.00**	0.66	1.00	**0.72**	0.39	1.00	yes	no	6 Feb 2020
Er-0340	2019	5	known age	10.24 (0.14)	4.39	0.15	0.69	0.38	0.89	**1.00**	0.63	1.00	0.04	0.01	0.51	yes	no	9 Apr 2020
Er-0340	2021	7	known age	11.36 (0.10)	4.32	0.15	0.59	0.26	0.79	**1.00**	0.64	1.00	0.02	0.00	0.38	yes	no	6 Jan 2022
Er-0376	2019	7	known age	10.7 (0.10)	4.33	0.15	0.55	0.24	0.78	**1.00**	0.64	1.00	0.01	0.00	0.36	yes	no	19 June 2020
Er-0318	2018	4	min age	9.99 (0.10)	4.67	0.14	0.55	0.13	0.89	**1.00**	0.52	1.00	0.00	0.00	0.43	yes	no	6 May 2019
Er-0332	2017	7	min age	10.58 (0.20)	5.08	0.14	0.46	0.02	0.95	**1.00**	0.55	1.00	0.00	0.00	0.49	no	n.a.	n.a.
Er-0301	2020	7	min age	10.69 (0.10)	4.97	0.13	0.31	0.01	0.90	**1.00**	0.60	1.00	0.00	0.00	0.55	yes	no	15 Aug 2021
Er-0344	2020	5	known age	9.54 (0.10)	3.66	n.a.	n.a.	n.a.	n.a.	0.77	0.04	1.00	n.a.	n.a.	n.a.	no	n.a.	n.a.
Er-0252	2016	2	known age	10.08 (0.28)	2.84	n.a.	n.a.	n.a.	n.a.	0.04	0.00	0.21	n.a.	n.a.	n.a.	no	n.a.	n.a.
Er-0318	2020	6	min age	9.99 (0.10)	4.34	n.a.	n.a.	n.a.	n.a.	**1.00**	0.65	1.00	n.a.	n.a.	n.a.	yes	no	5 Oct 2021
Er-0252	2021	7	known age	10.83 (0.10)	3.44	n.a.	n.a.	n.a.	n.a.	0.57	0.01	0.98	n.a.	n.a.	n.a.	yes	no	7 May 2022
Er-0375	2019	4	min age	9.69 (0.14)	4.42	n.a.	n.a.	n.a.	n.a.	**1.00**	0.58	1.00	n.a.	n.a.	n.a.	no	n.a.	n.a.
Er-0378	2020	2	min age	8.68 (0.10)	3.76	n.a.	n.a.	n.a.	n.a.	**0.87**	0.06	1.00	n.a.	n.a.	n.a.	yes	no	8 Aug 2021
Er-0207	2016	1	min age	9.23 (0.58)	2.02	n.a.	n.a.	n.a.	n.a.	0.01	0.00	0.16	n.a.	n.a.	n.a.	yes	no	30 June 2017
Er-0262	2016	2	known age	n.a.	3.64	n.a.	n.a.	n.a.	n.a.	**0.76**	0.03	1.00	n.a.	n.a.	n.a.	no	n.a.	n.a.
Er-0344	2018	3	known age	n.a.	4.12	n.a.	n.a.	n.a.	n.a.	**0.99**	0.38	1.00	n.a.	n.a.	n.a.	yes	no	25 June 2019
Er-0207	2017	2	min age	9.63 (0.68)	n.a.	0.15	n.a.	n.a.	n.a.	n.a.	n.a.	n.a.	0.02	0.00	0.37	no	n.a.	n.a.
Er-0076	2017	7	known age	9.12 (0.66)	n.a.	0.15	n.a.	n.a.	n.a.	n.a.	n.a.	n.a.	0.03	0.00	0.43	yes	no	6 Dec 2018
Er-0274	2016	4	known age	9.47 (0.24)	n.a.	0.15	n.a.	n.a.	n.a.	n.a.	n.a.	n.a.	0.01	0.00	0.37	no	n.a.	n.a.
Er-0318	2021	7	min age	10.45 (0.10)	n.a.	0.14	n.a.	n.a.	n.a.	n.a.	n.a.	n.a.	0.00	0.00	0.49	n.a.	n.a.	n.a.
Er-0318	2017	3	min age	9.99 (0.10)	n.a.	0.13	n.a.	n.a.	n.a.	n.a.	n.a.	n.a.	0.00	0.00	0.81	yes	no	19 June 2018
Er-0252	2020	6	known age	10.83 (0.10)	n.a.	0.15	n.a.	n.a.	n.a.	n.a.	n.a.	n.a.	0.01	0.00	0.36	yes	no	7 July 2021
Er-0358	2020	4	min age	9.25 (0.10)	n.a.	0.15	n.a.	n.a.	n.a.	n.a.	n.a.	n.a.	0.01	0.00	0.36	no	n.a.	n.a.
mature females (with fP4m and W50)																		
Er-0018	2019	20	min age	12.03 (0.17)	5.28	0.18	**1.00**	1.00	1.00	**1.00**	0.45	1.00	**0.99**	0.93	1.00	yes	no	16 Feb 2020
Er-0323	2019	12	min age	11.86 (0.41)	4.85	0.17	**1.00**	0.98	1.00	**1.00**	0.48	1.00	**0.93**	0.77	1.00	yes	no	22 June 2020
Er-0019	2017	21	min age	12.35 (1.40)	4.56	0.16	**0.96**	0.86	1.00	**1.00**	0.54	1.00	0.58	0.26	0.99	yes	no	30 July 2018
Er-0041	2017	17	min age	11.52 (0.14)	4.95	0.16	**0.96**	0.80	1.00	**1.00**	0.59	1.00	0.12	0.03	0.76	yes	no	9 Jan 2018
Er-0011	2018	17	known age	11.7 (0.14)	5.15	0.15	**0.96**	0.75	1.00	**1.00**	0.51	1.00	0.07	0.01	0.62	yes	no	6 May 2019
Er-0030	2019	19	min age	12.39 (0.14)	4.39	0.16	**0.86**	0.67	0.99	**1.00**	0.63	1.00	0.29	0.09	0.94	yes	no	21 June 2020
Er-0018	2020	21	min age	12.03 (0.17)	4.56	0.15	0.73	0.39	0.94	**1.00**	0.54	1.00	0.02	0.00	0.38	yes	no	22 May 2021
Er-0011	2017	16	known age	11.7 (0.14)	5.01	0.14	0.73	0.12	0.98	**1.00**	0.62	1.00	0.00	0.00	0.42	yes	no	6 May 2018
Er-0268	2018	13	min age	12.32 (0.22)	4.03	0.16	0.53	0.22	0.80	**0.98**	0.27	1.00	0.30	0.09	0.94	yes	no	13 Aug 2019
Er-0211	2017	22	min age	14.73 (0.51)	4.14	0.14	0.42	0.09	0.66	**0.99**	0.40	1.00	0.00	0.00	0.42	yes	no	8 Aug 2018
Er-0001	2018	16	known age	13.2 (0.44)	3.72	0.12	0.22	0.02	0.88	**0.82**	0.05	1.00	0.00	0.00	0.96	no	n.a.	n.a.
Er-0046	2020	34	min age	12.83 (0.10)	4.02	0.14	0.32	0.06	0.58	**0.97**	0.25	1.00	0.00	0.00	0.46	no	n.a.	n.a.
Er-0046	2016	30	min age	11.99 (0.30)	4.30	0.13	0.36	0.02	0.72	**1.00**	0.59	1.00	0.00	0.00	0.75	no	n.a.	n.a.
Er-0041	2018	18	min age	11.52 (0.14)	3.84	0.14	0.23	0.03	0.63	**0.92**	0.10	1.00	0.00	0.00	0.45	yes	no	9 June 2019
Er-0245	2017	21	min age	13.83 (0.22)	4.71	0.14	0.40	0.03	0.86	**1.00**	0.51	1.00	0.00	0.00	0.52	yes	no	8 Aug 2018
Er-0268	2019	14	min age	13.65 (0.48)	3.87	0.15	0.27	0.06	0.50	**0.93**	0.11	1.00	0.07	0.01	0.61	no	n.a.	n.a.
Er-0323	2017	10	min age	11.86 (0.41)	4.71	0.13	0.39	0.02	0.87	**1.00**	0.51	1.00	0.00	0.00	0.48	yes	no	20 Aug 2018
Er-0333	2018	15	known age	14.58 (0.49)	3.84	0.15	0.23	0.04	0.48	**0.91**	0.10	1.00	0.03	0.00	0.42	yes	no	26 July 2019
Er-0323	2021	14	min age	12.32 (0.10)	3.47	0.13	0.08	0.00	0.81	0.61	0.01	0.98	0.00	0.00	0.54	yes	no	18 June 2022
Er-0004	2021	25	min age	12.24 (0.14)	3.55	0.17	0.10	0.01	0.35	0.69	0.02	0.99	**0.93**	0.76	1.00	yes	no	21 July 2022
Er-0018	2021	22	min age	12.88 (0.10)	3.53	0.15	0.05	0.01	0.22	0.67	0.02	0.99	0.04	0.01	0.51	yes	no	24 May 2022
Er-0030	2020	20	min age	12.39 (0.14)	3.27	0.14	0.03	0.00	0.56	0.35	0.00	0.88	0.00	0.00	0.45	yes	no	9 Oct 2021
Er-0355	2017	8	min age	12.35 (0.77)	4.96	0.12	0.10	0.00	0.89	**1.00**	0.59	1.00	0.00	0.00	0.99	no	n.a.	n.a.
Er-0265	2018	6	min age	12.16 (0.42)	2.30	0.15	0.00	0.00	0.14	0.01	0.00	0.12	0.03	0.00	0.41	no	n.a.	n.a.
Er-0096	2016	14	min age	11.89 (0.14)	2.60	0.18	0.00	0.00	0.00	0.03	0.00	0.16	**1.00**	0.95	1.00	no	n.a.	n.a.
mature females (with fP4m only)																		
Er-0245	2016	20	min age	n.a.	6.11	n.a.	n.a.	n.a.	n.a.	**1.00**	0.01	1.00	n.a.	n.a.	n.a.	yes	no	24 Sep 2017
Er-0096	2021	19	min age	11.89 (0.14)	5.49	n.a.	n.a.	n.a.	n.a.	**1.00**	0.27	1.00	n.a.	n.a.	n.a.	n.a.	n.a.	n.a.
Er-0011	2016	15	known age	11.7 (0.14)	5.30	n.a.	n.a.	n.a.	n.a.	**1.00**	0.44	1.00	n.a.	n.a.	n.a.	yes	no	16 Sep 2017
Er-0014	2019	24	known age	11.97 (0.10)	5.00	n.a.	n.a.	n.a.	n.a.	**1.00**	0.62	1.00	n.a.	n.a.	n.a.	yes	no	22 June 2020
Er-0333	2019	16	known age	n.a.	4.62	n.a.	n.a.	n.a.	n.a.	**1.00**	0.52	1.00	n.a.	n.a.	n.a.	yes	no	27 Aug 2020
Er-0265	2017	5	min age	12.16 (0.42)	4.34	n.a.	n.a.	n.a.	n.a.	**1.00**	0.65	1.00	n.a.	n.a.	n.a.	yes	no	8 Aug 2018
Er-0018	2017	18	min age	n.a.	4.28	n.a.	n.a.	n.a.	n.a.	**1.00**	0.55	1.00	n.a.	n.a.	n.a.	yes	no	31 May 2018
Er-0030	2018	18	min age	12.39 (0.14)	4.24	n.a.	n.a.	n.a.	n.a.	**0.99**	0.50	1.00	n.a.	n.a.	n.a.	yes	no	7 Jan 2019
Er-0072	2020	34	min age	12.79 (0.10)	4.20	n.a.	n.a.	n.a.	n.a.	**0.99**	0.46	1.00	n.a.	n.a.	n.a.	yes	no	23 Aug 2021
Er-0323	2020	13	min age	12.32 (0.10)	4.13	n.a.	n.a.	n.a.	n.a.	**0.99**	0.40	1.00	n.a.	n.a.	n.a.	yes	no	6 Apr 2021
Er-0018	2018	19	min age	12.03 (0.17)	4.09	n.a.	n.a.	n.a.	n.a.	**0.98**	0.36	1.00	n.a.	n.a.	n.a.	yes	no	6 May 2019
Er-0014	2020	25	known age	11.97 (0.10)	4.06	n.a.	n.a.	n.a.	n.a.	**0.98**	0.32	1.00	n.a.	n.a.	n.a.	no	n.a.	n.a.
Er-0014	2016	21	known age	n.a.	3.93	n.a.	n.a.	n.a.	n.a.	**0.95**	0.15	1.00	n.a.	n.a.	n.a.	yes	no	7 June 2017
Er-0076	2019	9	known age	9.12 (0.66)	3.73	n.a.	n.a.	n.a.	n.a.	**0.83**	0.05	1.00	n.a.	n.a.	n.a.	yes	no	15 June 2020
Er-0362	2018	14	min age	12.35 (0.41)	3.72	n.a.	n.a.	n.a.	n.a.	**0.83**	0.05	1.00	n.a.	n.a.	n.a.	n.a.	n.a.	n.a.
Er-0019	2018	22	min age	n.a.	3.29	n.a.	n.a.	n.a.	n.a.	0.38	0.00	0.90	n.a.	n.a.	n.a.	yes	no	6 May 2019
Er-0245	2018	22	min age	n.a.	3.27	n.a.	n.a.	n.a.	n.a.	0.34	0.00	0.88	n.a.	n.a.	n.a.	no	n.a.	n.a.
Er-0076	2021	11	known age	10.7 (0.10)	3.08	n.a.	n.a.	n.a.	n.a.	0.19	0.00	0.65	n.a.	n.a.	n.a.	yes	no	6 Jan 2022
Er-0264	2016	19	min age	n.a.	2.88	n.a.	n.a.	n.a.	n.a.	0.07	0.00	0.29	n.a.	n.a.	n.a.	yes	no	26 June 2017
Er-0030	2016	16	min age	n.a.	2.81	n.a.	n.a.	n.a.	n.a.	0.05	0.00	0.21	n.a.	n.a.	n.a.	yes	no	26 June 2017
Er-0010	2018	10	min age	12.05 (0.66)	2.84	n.a.	n.a.	n.a.	n.a.	0.04	0.00	0.21	n.a.	n.a.	n.a.	yes	no	29 June 2019
Er-0287	2017	21	min age	11.2 (0.73)	2.83	n.a.	n.a.	n.a.	n.a.	0.04	0.00	0.20	n.a.	n.a.	n.a.	no	n.a.	n.a.
Er-0376	2021	9	known age	10.7 (0.10)	2.35	n.a.	n.a.	n.a.	n.a.	0.01	0.00	0.12	n.a.	n.a.	n.a.	yes	no	15 July 2022
mature females (with W50 only)																		
Er-0332	2019	9	min age	10.58 (0.20)	n.a.	0.17	n.a.	n.a.	n.a.	n.a.	n.a.	n.a.	**0.93**	0.77	1.00	no	n.a.	n.a.
Er-0011	2019	18	known age	11.7 (0.14)	n.a.	0.17	n.a.	n.a.	n.a.	n.a.	n.a.	n.a.	**0.92**	0.74	1.00	yes	no	7 June 2020
Er-0268	2016	11	min age	12.32 (0.22)	n.a.	0.17	n.a.	n.a.	n.a.	n.a.	n.a.	n.a.	**0.89**	0.65	1.00	yes	no	13 July 2017
Er-0276	2018	20	min age	12.33 (0.42)	n.a.	0.17	n.a.	n.a.	n.a.	n.a.	n.a.	n.a.	**0.85**	0.57	1.00	yes	no	22 July 2019
Er-0001	2016	14	known age	13.21 (0.35)	n.a.	0.16	n.a.	n.a.	n.a.	n.a.	n.a.	n.a.	0.62	0.30	0.99	yes	no	8 Sep 2017
Er-0284	2016	15	known age	12.74 (0.33)	n.a.	0.16	n.a.	n.a.	n.a.	n.a.	n.a.	n.a.	0.21	0.05	0.90	no	n.a.	n.a.
Er-0004	2020	24	min age	12.51 (0.10)	n.a.	0.16	n.a.	n.a.	n.a.	n.a.	n.a.	n.a.	0.16	0.04	0.85	yes	no	7 July 2021
Er-0246	2016	13	known age	11.95 (0.33)	n.a.	0.15	n.a.	n.a.	n.a.	n.a.	n.a.	n.a.	0.09	0.02	0.71	yes	no	6 Nov 2021
Er-0091	2018	21	min age	13.24 (0.77)	n.a.	0.15	n.a.	n.a.	n.a.	n.a.	n.a.	n.a.	0.06	0.01	0.58	yes	no	8 Sep 2019
Er-0043	2016	32	min age	12.22 (0.33)	n.a.	0.15	n.a.	n.a.	n.a.	n.a.	n.a.	n.a.	0.04	0.01	0.52	yes	no	25 Sep 2017
Er-0043	2017	33	min age	12.34 (0.24)	n.a.	0.15	n.a.	n.a.	n.a.	n.a.	n.a.	n.a.	0.03	0.00	0.40	no	n.a.	n.a.
Er-0001	2017	15	known age	13.2 (0.44)	n.a.	0.15	n.a.	n.a.	n.a.	n.a.	n.a.	n.a.	0.03	0.00	0.38	yes	no	9 June 2018
Er-0011	2020	19	known age	11.7 (0.14)	n.a.	0.15	n.a.	n.a.	n.a.	n.a.	n.a.	n.a.	0.02	0.00	0.38	yes	no	23 Aug 2021
Er-0054	2018	20	min age	12.54 (0.41)	n.a.	0.15	n.a.	n.a.	n.a.	n.a.	n.a.	n.a.	0.02	0.00	0.36	no	n.a.	n.a.
Er-0091	2019	22	min age	14.91 (0.52)	n.a.	0.15	n.a.	n.a.	n.a.	n.a.	n.a.	n.a.	0.01	0.00	0.37	no	n.a.	n.a.
Er-0076	2020	10	known age	10.63 (0.10)	n.a.	0.15	n.a.	n.a.	n.a.	n.a.	n.a.	n.a.	0.01	0.00	0.40	yes	no	15 June 2021
Er-0041	2016	16	min age	11.52 (0.14)	n.a.	0.15	n.a.	n.a. n.a.	n.a.	n.a.	n.a.	n.a.	0.01	0.00	0.40	yes	no	10 May 2017
Er-0041	2020	20	min age	11.52 (0.14)	n.a.	0.15	n.a.	n.a.	n.a.	n.a.	n.a.	n.a.	0.01	0.00	0.40	no	n.a.	n.a.
Er-0336	2016	7	min age	12.53 (0.20)	n.a.	0.14	n.a.	n.a.	n.a.	n.a.	n.a.	n.a.	0.01	0.00	0.40	yes	no	24 Sep 2017
Er-0323	2018	11	min age	11.86 (0.41)	n.a.	0.14	n.a.	n.a.	n.a.	n.a.	n.a.	n.a.	0.00	0.00	0.41	yes	no	7 Feb 2019
Er-0352	2019	17	min age	12.68 (0.46)	n.a.	0.14	n.a.	n.a.	n.a.	n.a.	n.a.	n.a.	0.00	0.00	0.46	yes	no	30 July 2020
Er-0243	2019	9	min age	12.54 (0.1)	n.a.	0.14	n.a.	n.a.	n.a.	n.a.	n.a.	n.a.	0.00	0.00	0.45	yes	no	7 Apr 2020
Er-0211	2018	23	min age	15.56 (0.51)	n.a.	0.13	n.a.	n.a.	n.a.	n.a.	n.a.	n.a.	0.00	0.00	0.49	yes	no	19 Aug 2019
Er-0243	2020	10	min age	12.54 (0.10)	n.a.	0.13	n.a.	n.a.	n.a.	n.a.	n.a.	n.a.	0.00	0.00	0.49	no	n.a.	n.a.
Er-0030	2021	21	min age	12.94 (0.10)	n.a.	0.13	n.a.	n.a.	n.a.	n.a.	n.a.	n.a.	0.00	0.00	0.77	n.a.	n.a.	n.a.
Er-0336	2017	8	min age	12.53 (0.20)	n.a.	0.13	n.a.	n.a.	n.a.	n.a.	n.a.	n.a.	0.00	0.00	0.88	no	n.a.	n.a.
Er-0010	2017	9	min age	10.8 (0.72)	n.a.	0.12	n.a.	n.a.	n.a.	n.a.	n.a.	n.a.	0.00	0.00	0.99	yes	no	31 May 2018

#### 3.1.2. Field-observed reproductive state relative to drone-based photogrammetry

**Figure 3.**
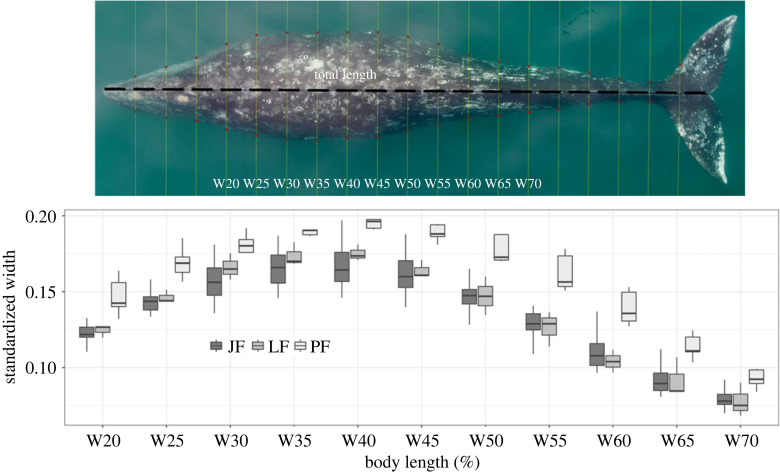
Comparison of body widths at 5% increments along total body length in female gray whales of known reproductive status from UAS-based photogrammetry (example photograph shown at top). Presumed pregnant (PF) whales are those resighted with a calf the year following sampling (blue), presumed non-pregnant juvenile females (JF; yellow) are immature females, and lactating females (LF; orange) are females sighted with a calf at the time of sampling; the latter two groups are presumed non-pregnant. Width measurements are standardized by total body length to provide a scale-invariant and unitless metric that allows for comparison across individuals.

Pregnant females (PF, *n* = 5) exhibited higher standardized width measurements along their body length as compared with the presumed non-pregnant groups (JF: *n* = 17 and LF: *n* = 3). Within the exploratory data analysis, we observed the largest separation between reproductive groups between 40% and 55% of TL (figure 3). In particular, W50 presented the best separation, with minimum overlap between PF (mean = 0.18, s.d. = 0.01) and presumed non-pregnant groups (JF: mean = 0.15, s.d. = 0.01 and LF: mean = 0.15, s.d. = 0.01; table 1). At the individual level, females resighted in multiple years with different presumed reproductive statuses (*n* = 4) exhibited greater W50 values when presumed pregnant (PF) as compared with when presumed non-pregnant (LF and MF; figure 1, bottom row). One individual, Er-0271, was sampled only as PF; although no drone observations are available from this individual in a different reproductive status, her W50 values were similar to other PF individuals. Results from the Monte Carlo ANOVAs comparing W50 by female demographic unit (JF, LF, PF) indicates that PF (mean = 0.178, 95% HPDI = 0.157, 0.200) had significantly larger W50 than JF (mean = 0.150, 95% HPDI = 0.137, 0.165) and LF (mean = 0.147, 95% HPDI = 0.118, 0.174) (electronic supplementary material, figure S1). Based on these exploratory analyses, we used W50 in subsequent analyses.

### 3.2. Mixture models

#### 3.2.1. Comparing mixture models' performances

**Figure 4.**
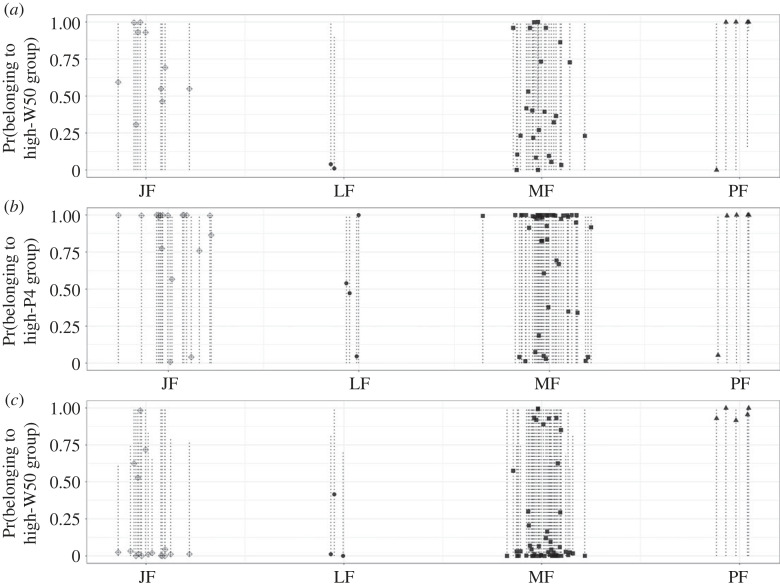
Probability of being pregnant based on (*a*) Model 1: two-component multivariate normal mixture model with the log-transformed fP4m (faecal progestogen metabolites) and W50 (width at 50% of total body length); (*b*) Model 2: two-component normal mixture model using the log-transformed fP4m only; and (*c*) Model 3: two-component normal mixture model using W50 only. Dots indicate the mean probability of belonging to the component with high fP4m and high W50 for Model 1, and the median probability of belonging to the component with high fP4m or high W50 for Models 2 and 3 based on bootstrapping results. Vertical dashed lines indicate the 95% confidence intervals. Juvenile female (JF), lactating female (LF), mature female (MF), pregnant female (PF).

Overall, the three different models produced large confidence intervals around the probability of pregnancy assigned to each individual, illustrating the challenges of defining two components that accurately diagnose pregnant and non-pregnant states (figure 4). Although the interquartile ranges of both fP4m and W50 indicate that extreme probability values are uncommon (table 2), they influence the models’ performances, particularly due to the high overlap we observed in the range of fP4m for the two groups (Models 1 and 2). Model 3 (only using W50) was better able to separate the two groups and performed well at classifying individuals of known reproductive status, particularly the JF group (figure 4 and table 2).

The performance of the three models was assessed based on their ability to correctly classify whales of known reproductive status, i.e. presumed pregnant (PF) and presumed non-pregnant (LF and JF; table 3) females. Individual gray whales were predicted to belong to the pregnant groups when the mean probability assigned by the model was equal to or higher than 75%. Models 1 and 3 performed with reasonably low misclassification rate (less than 25%). However, Model 2, in which fP4m was the only variable used to classify pregnancy presented a higher misclassification rate (67%). The two models that include fP4m (Models 1 and 2) also exhibited significantly lower ability to correctly classify PF (true positive rate = 80%) compared with the model with only W50 (Model 3), in which all presumed pregnant females were correctly classified as pregnant. Hence, Model 3 also had a 0% false negative rate. Model 3 also had the lowest false positive rate, with an 8% probability of misclassifying non-pregnant individuals as pregnant. Hence, Model 3 that applied only W50 in a two-component mixture Model had the highest accuracy (95%) and lowest misclassification rate (8%).

**Table 3.** Model performance comparisons. The models are compared in their ability to correctly classify individuals of known reproductive status; for this purpose, we considered all females with a probability of pregnancy greater than or equal to 75% to be classified as pregnant. TP, true positive, number of individuals classified as pregnant when they were presumed to be pregnant; TN, true negative, number of individuals classified as not pregnant when presumed not pregnant; FN, false negative, number of individuals classified as not pregnant when presumed pregnant; FP, false positive, number of individuals classified as pregnant when presumed not pregnant; and total, total number of individuals used for evaluating the models' performances; PF, females seen with a calf the year after sampling; LF, females seen with a calf the year of sampling; JF, immature female.

performance	MODEL			formula
	1	2	3	
accuracy	71%	36%	92%	(TP + TN)/Tot
misclassification rate	29%	64%	8%	(FN + FP)/Tot
true (+) rate	80%	80%	100%	TP/(TP + FN)
false (−) rate	20%	20%	0%	FN/(FN + TP)
true (−) rate	67%	26%	90%	TN/(TN + FP)
false (+) rate	33%	74%	10%	FP/(FP + TN)
presumed pregnant	5	5	5	PF
presumed non-pregnant	12	23	20	LF + JF
total	17	28	25	PF + LF + JF

*Post hoc* assessment of presumed non-pregnant individuals that were misclassified as pregnant (table 2) indicates that two immature females (JF; Er-0358 and Er-0377 in 2019) were classified as pregnant by all three models and two other JFs (Er-252 and Er-318 in 2019) were classified as pregnant by two models (Models 1 and 2). Owing to incomplete sighting histories that limit our knowledge of these whales' true age and maturity status, and potential for pregnancy loss, it is challenging to determine the accuracy of these pregnancy classifications. However, one of these two JFs (Er-0377 in 2019, consistently classified as pregnant by all three models) had a known age (age = 5 years) and was resighted in early February of the following year without a calf, whereas the other whale (Er-358 in 2019) had a minimum age (min age = 3) and was also resighted the following year with no calf in late August. The high probability of pregnancy for these individuals was influenced primarily by elevated levels of fP4m, but also by moderately high values of W50 (table 2). Of the other two JFs classified with high probability of pregnancy by both Models 1 and 2, one had a minimum age of 5 (Er-0318 in 2019) and was resighted without a calf in the following year in early June, and the other (Er-252 in 2019) had a known age (age = 5) and was resighted with no calf in March of the following year. These individuals presented moderately elevated W50 and elevated fP4m (table 2). In addition, 13 other JFs were classified as pregnant only by Model 2 (table 2), but due to the low true positive rate of Model 2 (table 3) we deem these unreliable classifications. Lastly, one LF (Er-0014 in 2018) was classified as pregnant with high probability by Model 3, yet this individual was resighted with no calf the following year in July. The estimated high probability of pregnancy for this individual was influenced primarily by the whale's moderately high W50. One additional LF was also classified as pregnant with high probability by Model 2 (Er-0019 in 2020) based on relatively high fP4m and was resighted the following year with no calf (table 2). However, given the high overlap observed in the ranges of fP4m in these two groups (PF and presumed non-pregnant JF and LF), these classifications may be unreliable.

#### 3.2.2. Application of the models to assign probability of pregnancy to the mature females of unknown reproductive status

Mature females that were not observed with a calf the following year (MF), and from which we obtained fP4m and/or W50 data, presented fP4m concentrations and W50 measurements that fell both within and outside of the known-pregnant ranges. Two individuals (Er-0018 and Er-0323 in 2019, table 2) were consistently assigned high pregnancy probability by all three models. The multivariate model (Model 1) classified 6 out of 25 MFs as pregnant with probability (i.e. **P(p)** > 75%). By contrast, the univariate model based only on fP4m (Model 2), produced the largest number of MFs classified as pregnant with high probability, with 34 individuals out of a total of 48 assigned a pregnancy probability greater than 75%. This result probably reflects the high misclassification and false positive rate associated with this model (table 3). The univariate model using just W50 (Model 3), and the best overall performance (table 3) predicted a total of 8 out of 52 MFs to be pregnant with high probability (i.e. **P(p)** > 75%). These predicted pregnancies occurred in MFs observed in 2016 (*n* = 2), 2018 (*n* = 1), 2019 (*n* = 4) and 2021 (*n* = 1).

## 4. Discussion

Our analysis of a 6-year-long dataset of faecal hormone metabolites, drone-based photogrammetry and sightings revealed the strengths of drone-based body morphology and weaknesses of fP4m (using the specific assay antibody used here) for non-invasive pregnancy diagnosis in PCFG gray whales. The use of the W50 metric in the univariate mixture model (Model 3) successfully separated PF and non-PF females, while the high variability of fP4m limited its application to identify pregnancy accurately (Models 1 and 2). When comparing model performance validated by individuals of known reproductive status (table 3), the univariate model that used only W50 (Model 3) resulted in high accuracy (92%) with a low misclassification rate (8%). The multivariate approach using both W50 and fP4m (Model 1) is comparatively less accurate than Model 3, while the univariate approach using only fP4m (Model 2) resulted in the lowest accuracy with the highest false positive rate. Hence, it is clear that the lack of precision associated with the fP4m variable negatively influenced the performance of the multivariate approach.

Lemos *et al*. [35] used drone-based photogrammetry of PCFG gray whales to measure and compare body condition between individuals, using the body area index (BAI, [33]) metric and found that PFs (*n* = 3) were the demographic class with the highest BAI. Our finding that PFs are significantly wider than non-pregnant females align with these initial results presented in Lemos *et al.* [22,35]. Despite a low sample size of confirmed PF (*n* = 5), the body width at 50% of TL (W50) satisfactorily discriminated pregnant from non-pregnant females, and Model 3 provided a useful analytical approach to assign pregnancy probability. In addition to the five confirmed pregnant females, we identified eight MF of unknown reproductive status and two JF with high pregnancy probability (greater than 75%) using Model 3. Although the two JF would traditionally be classified as sexually immature, observational data from the western North Pacific gray whale population indicates that the maturity age for gray whales could be as young as 5 years [61]; hence, it is possible that these two individual JF cases corresponded to true pregnancies. Thus, the use of W50 in Model 3 allows us to provisionally increase the total number of PF in this 6-year study, which might imply higher pregnancy rates than estimated by calf sighting only.

Among the PF confirmed with calf resighting, two were observed in 2016, and one each year in 2017, 2018 and 2019 (table 2). Of the eight putative MF pregnancies identified by Model 3, most occurred in 2019 (50%; *n* = 4 of 8), followed by 2021 (25%; *n* = 1 of 4), 2016 (22%; *n* = 2 of 9) and 2018 (11%; *n* = 1 of 11). Out of these eight MF, four had both morphometric and hormone data. Among these four individuals, two were also classified with a high probability of pregnancy by Models 1 and 2 (table 2). It is possible that these whales were true pregnant but lost their calf before resighting, or the pregnancies did not reach full term (see conclusion). In 2017 and 2020, no MF was classified as pregnant by this model. Current sample sizes of MF are too small to detect any patterns in the annual variability in pregnancy rates; however, other baleen whale studies [37,54,67,68] noted that the proportion of pregnant females correlates with larger oceanographic fluctuations that influence prey availability. Continued long-term research programmes with targeted sampling towards the end of the season can improve sample size and allow increased exploration of temporal patterns in pregnancy rates and correlations with environmental conditions.

Interestingly, the ENP reproductive rates estimated based on calf production [69] show declines corresponding to the current UME (2019–present) [70] and a previous UME (1999–2000), with declines in the estimated abundance also occurring during these periods and in 2007–2010 [47,69]. Application of the Model 3 with aerial morphometric data (W50) collected from ENP gray whales during the southbound migration would provide an opportunity to assess pregnancy rates and estimate calf loss once data are compared with calf count data collected during the northbound migration. Such derived data could improve population models and evaluation of drivers of UMEs.

Our inability to reliably use fP4m to diagnose pregnancy in this study is probably a consequence of (i) the timing of faecal collection with respect to the gestation period, which could explain the high overlap in the fP4m range between the PF and presumed not PF groups, (ii) faecal consistency of this species and/or sample collection method, (iii) our low sample size, and/or (iv) the specific assay antibody used. The high overlap in fP4m levels between reproductive groups of female PCFG gray whales could be attributed to the timing of sampling, which falls between the first six to eight months of pregnancy when the fP4m concentrations may still be low, as gestation in gray whale females lasts approximately 13 months [7,48] and progesterone levels typically increase steadily throughout gestation [71]. While Lemos *et al*. [22] found slightly but statistically significantly elevated levels of fP4m in pregnant PCFG females (*n* = 4) as compared with the other demographic groups [22], studies of fP4m in other cetacean species have documented orders of magnitude higher levels in PFs as compared with non-pregnant groups (e.g. [27–30]). In addition, consistency of gray whale faecal samples may impose constraints on utility of fP4m in this species. PCFG gray whales typically produce faeces that consists of fine, unbound particles that rapidly disperse in the water column, forming a fast-sinking ‘faecal plume’ that poses challenges to the recovery of adequate amount of sample for representative hormone quantifications, as faecal steroid metabolites are probably unevenly distributed in the faeces [23]. Hence our opportunistic sample collection process may introduce variability based on whether we collect a hormone ‘hot spot’ or not [23]. Evidence for this possibility is the relatively high coefficient of variation between samples collected from the same individual on the same day ranging from 0.36 to 65.

Although our sample size of PF was low (*n* = 5), it is comparable to previous studies that successfully applied fP4m to distinguish pregnancy in baleen whales (4-year study of North Atlantic right whales produced 3 PF [27]; 13-year study of North Atlantic right whales produced 14 PF [28]; 2-year study of humpback whales produced 4 PF [29]). In the North Atlantic right whale and humpback whale studies, fP4m analysis provided much clearer separation of pregnant from non-pregnant females than we document here for gray whales. These marked differences in the utility of fP4m data for pregnancy diagnosis in different studies may be due to species-specific differences in progesterone metabolization in the gut, and/or to the different assay antibodies used in these various studies (prior studies on other large whales used an antibody that is no longer commercially available). Progesterone in terrestrial mammals is metabolized in the gut to up to 18 different faecal metabolites [18,72], with progesterone itself often no longer detectable at all, and the proportion and identities of the metabolites are highly species-specific and, sometimes, even population-specific (varying, for example, with diet, digestive enzymes and gut microbiota) [73]. The major progesterone faecal metabolites have not been identified for any species of large whale, because the necessary validations (infusions of radiolabelled progesterone, or ‘challenge’ experiments with infusions of hypothalamic and pituitary hormones) are not logistically feasible. Further, any given progesterone antibody typically cross-reacts with only some faecal metabolites of progesterone, such that different antibodies—even if originally raised against the same parent hormone, progesterone—can produce quite divergent data from faecal samples of the same species. Small faecal masses in this study prevented the comparison of multiple antibodies, but other commercial antibodies for faecal progesterone metabolites do now exist and could be tested. Thus, we suggest that other antibodies and potentially other hormone quantification methods (e.g. liquid chromatography with tandem mass spectrometry) also be explored for this species, as it is possible that another fP4m quantification method might yield improved data for pregnancy diagnosis in gray whales. The unique characteristics of our study system, i.e. high individual resighting rates, with over 30 years of sighting history and the non-invasive nature of the faecal hormone approach that allows us to obtain multiple samples across and within season, provide an advantage over other study systems to enable continued development of this technique, including testing alternative hormones, specific antibodies, or alternative determination and quantification techniques.

## 5. Conclusion

All species of baleen whales were heavily depleted by commercial whaling during the past several centuries, and today are exposed to multiple anthropogenic stressors (e.g. entanglement in fishing gear, vessel strikes, shipping noise, boat interactions, etc.; [74]). These stressors may cause direct mortality, but more frequently they led to indirect sublethal effects on individuals, such as long-term changes in health and reproduction that can ultimately result in impacts at the population level [75]. Moreover, detecting changes in a population's reproductive trend might indicate wider shifts in the marine ecosystem. For example, reproductive failure in large whales has been linked to changing environmental conditions [76], declines in prey availability [68,77,78], entanglements in fishing gear [79–82] and naturally occurring toxins [83]. Fecundity estimates for large whales are usually based on calf sightings, but such estimates have long been suspected to be underestimates of actual pregnancy rate, since some pregnancy loss and calf mortality presumably can occur before calves are sighted. Thus, calf sightings data may underestimate the reproductive capacity of the population, and may also underestimate the impact of potentially important natural and anthropogenic stressors, especially any that may disproportionately affect pregnant females or young calves. Therefore, researchers have attempted to diagnose pregnancy using several approaches [66], including quantification of hormone concentrations in faeces [27–29], blubber [8,9,11,84] and respiratory vapour [85]. Of these sample types, faecal analysis has the benefits of being completely non-invasive with minimal disturbance to the animal during sample collection and is now widely employed for studies of stress and reproductive physiology in terrestrial and aquatic wildlife [20,86]. However, the correct interpretation of faecal hormone data can be complex and requires careful validation, both analytically and biologically, when implementing with a new species or a new quantification technique [5,15,29]. Our assessment of the utility of applying the faecal hormone techniques and drone-based photogrammetry for determining pregnancies in the PCFG gray whales highlights the need for further testing and validation of faecal hormone methods.

Interestingly, the univariate mixture model using only a morphometric measurement, length standardized body width at the 50% of the body length (W50), proved to be reliable in determining pregnancies in this population. Even with a small sample size of confirmed pregnancies we were able to apply these methods to accurately classify PFs. Thus, given that drones are becoming increasingly common in whale research programmes, we encourage research teams to evaluate this morphometric approach to diagnose pregnancy in other whale species and in other gray whale populations, e.g. the ENP or the endangered western North Pacific gray whale population. As demonstrated in our study, this non-invasive approach to pregnancy identification has the potential to improve our ability to monitor variation in important, yet challenging to estimate, baleen whale population metrics of pregnancy and calf loss rates.

## Data Availability

The processed datasets generated for this study and relevant analysis code are available on the Figshare Digital Repository https://doi.org/10.6084/m9.figshare.22573231 [87]. The data are provided in electronic supplementary material [88].
